# Report of Surgical Cases, Treated at the City Hospital during the Summer Term

**Published:** 1860-08

**Authors:** George K. Amerman

**Affiliations:** Attending Surgeon


THE CHICAGO
### MEDICAL JOURNAL.

VOL. III. August, 1860. NO. 8.

### Original Communications.

### ARTICLE 1.

### REPORT OF SURGICAL CASES

TREATED AT THE CITY HOSPITAL DURING THE SUMMER TERM OF INSTRUCTION, BY GEORGE K. AMERMAN, M. D., ATTENDING SURGEON.

(Continued from the July No. of Chicago Medical Journal.)

CASE 5.—*Stone in the Bladder—Operation of Lithotomy—Recovery.*

August Schmidt, aged eight years, born in Germany; been in this country three years. Admitted into the City Hospital March 15th, 1860. No hereditary tendency to disease traceable; parents both living; The patient has always enjoyed good health until about ten months ago, at which time he began to suffer from pain and difficulty in urinating. At first, for several months, he concealed his sufferings, and nothing was known of it till September 1859, when it became so severe as to oblige him to discontinue his attendance at school and remain in his room most of the time. Several physicians were consulted, none of whom recognized the real trouble, or afforded any permanent relief.

I visited him, at his home, for the first time, in February, 1860. I found him in bed, greatly emaciated, having no appetite, and at times suffering the most excrutiating pains. Aside from his general appearance, which indicated some serious constitutional trouble, the pain was the only marked symptom. This was paroxysmal, coming on every afternoon, lasting from two to four hours, then entirely disappearing until about midnight, when a second exacerbation would set in and continue till near morning. This regular recurrence of the pain was not invariable, the afternoon pain being occasionally absent, in which case the night exacerbation came much earlier. There was no apparent connection between these paroxysms of pain and urination. It was seated in the hypogastric region, the thighs, perineum, and particularly in the glans penis. The prepuce was elongated, the glans penis small, and the testacles drawn entirely out of the scrotum into the abdomen. He had learned, instinctively, to urinate whilst lying on his back with the legs and thighs closely flexed, and always assumed that position. He had never had any sudden stoppage of urine, had never been obliged to urinate oftener than three times a day, and never had suffered from hematuria. The urine contained an abundant deposit of the triple phosphate, mucous, and epithelium, but no pus or albumen. On using a sound, the presence of a calculus was readily ascertained, also its small size, and mobility. In sounding it was always necessary to administer an anesthetic. After repeated soundings, with invariably the same result, both by myself and my friend Dr. Ernst Schmidt, I advised his removal to the City Hospital and the operation of lithotomy. This was readily assented to by the parents, and March 20th, the time fixed for the operation. The only preparatory treatment consisted of a cathartic administered the night previous.

The operation was performed in presence of the class attending the summer course, and several physicians of the city. The instruments used consisted of an ordinary scalpel, grooved staff, and lithotomy forceps. The staff, owing to the small size of the urethra, corresponded with a No. 7 sound. He was placed on a firm table, and sulph. ether given as an anesthetic. After the production of partial anesthesia, the bladder was injected with about four ounces of tepid water, the staff introduced and held in the mesial line, closely hooked against the pubes, and the limbs firmly held in the usual way. The action of the ether was slow and imperfect, nearly a pint being inhaled before its effects were sufficiently strong to commence the operation. The struggles of the patient during its inhalation, were also embarrassing, and delayed the operation for several minutes, besides causing the expulsion of the contents of the bladder. The old lateral method of operating was adopted in this case, and as it differed in no respect from the ordinary mode, requires no particular description. The bladder was found perfectly empty, the stone resting at its bas fond, and easily extracted. Not over one ounce of blood was lost during or after the operation. The patient was immediately removed to a bed, properly prepared to protect him from the contact of urine, an anodyne given, and the wound left entirely to itself. The stone was of small size, conical in shape, and exceedingly rough on its surface.

March 21.—Patient slept well last night; better than at any time during the last three months. Urine passes freely through the wound. No pain; pulse 140. Ordered emulicient drinks and small doses of digitalis.

March 22.—Doing well this morning. Slight tenderness over the hypogastric region; pulse 140. At noon the abdominal tenderness had increased, also considerable tympanitis. Gave opium freely, and applied warm fomentations. At midnight pulse 138; patient sleeping and perspiring freely.

March 23.—Pulse 130; tenderness considerably less; opium occasionally; fomentations as usual.

March 27—(Seventh day.)—All symptoms of peritonitis entirely disappeared. Urine passed per urethram for the first time last night. The greater portion, however, continues to discharge through the wound. From this time forth the daily notes of the case contain nothing of interest or importance. He was discharged cured May 23d, sixty-four days after the operation.

*Remarks.*—One feature in the symptoms of the above case, the paroxysmal character of the pain, is rather an uncommon accompaniment of this affection; otherwise it presents nothing unusual. The absence of hematuria, increased frequency of micturition, and sudden stoppage of urine, may all be accounted for by the absence of any organic disease of the genito-urinary organs. Nearly all the rational symptoms of stone, except the sudden stoppage of the flow of urine, depend on disease of the urinary organs, produced by the continued irritation of the calculus, and hence, in the absence of any such disease, there is, necessarily, an absence of all rational symptoms. The physical sign obtained by the sound, the click heard when a solid instrument is introduced into the bladder and brought in contact with the stone, can alone be depended upon in the diagnosis of calculus, and even it is not infallible. The operation of sounding, simple as it is, requires great care and considerable experience, to make it satisfactory or certain. It should never be prolonged beyond a reasonable length of time, serious and even fatal consequences having occasionally resulted from a protracted sounding. It should always be repeated several times before a positive opinion is rendered, or an operation advised. It should always be resorted to just before the operation, after the patient is on the table and every thing prepared, and if no calculus be then felt, the operation must be postponed. It, in connection with the rational signs, generally renders the diagnosis easy and certain, but when we remember that such men as Cheselden, Dupuytren, Roux, Crosse, Tyrrell, Cotta, Vacca, and others equally as eminent, have all cut into the bladder and found nothing, it cannot fail to make us exceedingly cautious.

The treatment of stone has, at the present time, narrowed itself down exclusively to two operations. *Lithotrity*, or the introduction of instruments especially made for that purpose, into the bladder, grasping the stone and crushing it, and *Lithotomy*, or the cutting operation, the incision of the bladder either through the abdomen, perineum, or rectum. Nearly every case of stone will admit of one or the other of the above operations. If the surgeon finds the urethra large, the urinary passages and bladder healthy, the calculus small and friable, he may recommend lithotrity, with a reasonable expectation of its being successful. But, if the calculus is large and dense, the urethra small, and the bladder irritable, lithotomy must have the preference. Every case should be carefully studied, and that course of treatment selected best calculated to meet the peculiarities of that particular case. Prejudices against, or preferences for, any particular operation or plan of operating, should never influence the surgeon to a course against the best interests and welfare of his patient. They are both attended with difficulties and dangers which should be fully understood by every one undertaking their performance. The difficulties may be, in part, overcome, but most of the dangers, are inherent to the operation, and cannot be avoided. One observation, in explanation of the long time the above case was under treatment after the operation, is, perhaps, necessary. The urine passed through the urethra for the first time on the seventh day after the operation, and continued to do so without entire interruption ever afterwards, at the same time the most of it was voided through the wound. This depended principally, on the patient's being stubborn and contrary, so that he would frequently allow the bladder to become immensely distended without any attempt or effort to urinate, naturally, and, whilst in this condition the adhesion of the wound would give way, and the water gush out through it. This condition of things existed for several weeks, in spite of all our efforts to the contrary, and finally resulted in a fistulæ, which added considerably to the time required for a complete cure. Under more favorable circumstances, we believe half the time could easily have been saved. No kind of dressings were ever used to the wound.

CASE 7.—*Railroad Injury—Amputation of the Thigh—Recovery.*

M. A. C., aged six years, was brought to the Hospital Feb. 17, 1860, with a severe injury of the right lower extremity, received about two hours previously. She states that while she was under a loaded freight car, endeavoring to get a piece of wood, the train started, and in her effort to escape her right limb was caught in such a way that one of the trucks passed entirely over it. The injuring extended from the ankle to the knee, lacerating all the soft parts, and fracturing both bones in several places. The blood vessels were so torn and twisted that when the part was kept quiet there was scarcely any bleeding. The foot was cold, pale, and insensible; the pulse nearly normal. There was no pain in the part, and as soon as the patient was placed in bed in a comfortable position, she sank into a quiet, natural sleep. The severity of the injury precluded any attempt to save the limb, and after obtaining the consent of the parents, it was decided to remove it immediately. Sulph. ether was given as an anesthetic which acted favorably without producing any unpleasant nausea or vomiting after the operation. The limb was amputated at about the middle of the thigh, the anterior and posterior flap method, not over two ounces of blood being lost during its performance. Simple dressing were applied and the case placed in charge of Dr. H. Durham during the night, with directions to give opium and brandy according to circumstances.

March 18.—During the night the patient has taken half an ounce of brandy and one grain of morphine. Reaction perfectly established this morning. Pulse 128; patient sleeping quietly from the effect of the anodyne; stump not disturbed.

March 19.—Patient slept the greater part of last night under the influence of an anodyne. Pulse 140; with considerable fever; stump looking finely; flaps united, throughout most of their extent; gave digitalis in small doses; simple dressings to the wound; anodyne at night.

March 23.—Constitutional excitement nearly subsided; wound discharging thin, unhealthy pus; flaps separated somewhat since last note; ordered quin. and iron; good diet, and porter. From this time onward the daily notes of the case contain nothing of interest or importance. The wound, owing to the gradual impairment of the general health of the patient healed slowly, and at the end of my term of service at the hospital was nearly as large as the palm of the hand. About one week ago, the last time I saw the case it had nearly cicatrised.

CASE 7.—*Railroad Injury—Amputation of the Leg—Gangrene of stump—Death on the 9th day.*

E. J. B. aged 37 years, born in England, was admitted into the City Hospital March 15th, near midnight, having received an injury of the left foot and ankle about four hours previously. The injury extended from the toes, on the dorsal surface of the foot, to two inches above the ankle joint, lacerating and completely destroying the greater portion of the integument, exposing the flexor tendons of the foot, separating and denuding the bones of the meta-tarsus, and opening the anterior surface of the ankle joint. There was no fracture or dislocation at the time of her admission. The shock of the injury, added to the fatigue of a long ride subsequent to its occurrence, and the loss of a large quantity of blood, had produced greater prostration than usual. She was placed in bed, the injured foot and leg covered with lint dipped in cold water, and brandy and opium given freely during the remainder of the night. In a few hours, reaction set in and became fully established early the following day (16th).

The amputation of the leg was at once recommended, consent given, and the operation performed at the afternoon clinic hour of the hospital, in presence of the class. Sulph. Ether was given as an anesthetic, the limb amputated in its upper third just below the tuberosity of the tibia, by the anterior and posterior flap method. Very little blood was lost during the operation, and that mostly venous. The flaps were brought together by interrupted sutures, and strips of adhesive plaster. A piece of lint soaked in water applied to the end of the stump, the whole confined with a roller bandage completed the dressings. The patient placed in bed, and brandy and opium given during the day and night.

March 17.—Patient rested a part of last night after taking one gr. morphia. During the latter part of the night she became very thirsty and drank a large quantity of water. Pulse 100, skin warm, no pain except an occasional twinge in the stump; stump not disturbed. Ordered pills of quin. and iron during the day, anodyne at night.

March 18.—Rested only the fore part of the night. Considerable sharp, shooting pain in the stump. Pulse 100, the flaps of the wound lying against each other without any adhesion of their surfaces or evidence of inflammation. A thin, reddish, serum ran out when the lint was removed, but not the slightest trace of suppuration. Several vesicles filled with same kind of material existed on the anterior flap and surface of the limb. Same dressings re-applied and in addition to the quin. and iron, ordered beef tea and porter.

March 20.—Pulse 112, skin very hot, patient restless, and suffering from pain in the stump; no appetite; stump swollen, of a purplish red color; vesicles scattered over it, and discharging a thin, unhealthy pus; continue same constitutional treatment. The stump was thoroughly cleansed by injecting a sol. of chlorid soda, then painted with a sol. iodine, the flaps dusted with pulv. cinchona and a roller applied.

March 22.—Patient beginning to show signs of exhaustion; no appetite, no pain, skin sometimes hot, sometimes covered with cold perspiration. The stump adematous and languid; the anterior flap dead and sloughing; continue same treatment with the addition of brandy.

March 25—(Ninth day.)—Patient moribund; unable to speak loud enough to be heard; skin cold and covered with perspiration; breath cold; no pain; stump emphysematous; blue lines running up the thigh; died at noon.

*Remarks.*—The two preceeding cases represent a class of injuries of frequent occurrence and of the first importance to the Surgeon. Railroad injuries closely resemble in their nature and tendencies gun-shot wounds. They are always followed by severe constitutional disturbance, and frequently, a much more serious local lesion than their first appearance indicates. When they are of such a nature as to call for amputation of the injured part, the operation is always a very serious one. Primary amputation, of the lower extremity, for railroad injury, is one of the most fatal operations in surgery. Primary amputation of the lower extremity, is always a serious undertaking. Of twenty-four cases of amputation of the thigh for injury recorded by the London surgeons, every one proved fatal. Of thirty-one cases of amputation of the leg, seventeen proved fatal, over one-half. The fatality of primary amputations for railroad injuries is determined in a great degree by the surrounding circumstances. The situation of the injury, its extent, the length of time after the accident, the amount of blood lost, the age, constitution and habits of the patient all exercise an important influence over the result of the operation. The causes of death are essentially the same as in other injuries. Shock, hemorrhage, erysipelas, and empyemia, are among the most common. The shock attending railroad injuries is always greater in proportion to the extent of the injury than in similar cases produced by other means. The hemorrage, like that of all lacerated and contused wounds is seldom of a serious character. It does not often happen that persons die of railroad injuries where the extremities are alone involved, either from shock or hemorrhage, but, when any of the internal organs are implicated, or the case not properly attended soon after the occurrence of the injury, the combined effects of the two, will frequently prove fatal in a few hours. Two such instances have come under my observation during the last year. Both happened in the night some distance from the city, and were not seen until several hours after their occurrence. During this time nothing was done to arrest the hemorrhage or bring on reaction and when the cases came under my charge, the prostration was so great that neither reacted or survived longer than twelve hours. In both, the injury was confined to the lower extremities.

Erysipelas, gangrene, and empyemia, are frequent occurrence after railroad injuries, and especially after amputations performed for their relief. The nature of the injury, the crushing and mangling of the parts, seems in some way, to predispose the system to each of the above accidents. Empyemia is especially apt to follow, and should always be anticipated and as far as possible guarded against. It generally manifests itself during the second week, sometimes in comparitively mild cases, and proves rapidly fatal. The treatment of these injuries, must depend on the nature, extent, and situation of the wound, the condition, constitution, age, and habits of the patient, and other circumstances surrounding the case. No particular plan is applicable in every case. They must be treated on general principles. In every doubtful case, an attempt should be made to save the limb, and, if unsuccessful, a secondary amputation resorted to, which is attended by a much more favorable result than the immediate removal of the part.

